# Clinical Spectrum and Outcomes of Typhoid Fever: A Retrospective Study

**DOI:** 10.7759/cureus.94621

**Published:** 2025-10-15

**Authors:** Zeeshan Shafqat, Saadia Kanwal, Abdullah Yousaf, Afsah Shafqat, Zarghoona Kamal, Muhammad Ismail Zahid, Saima Shabir

**Affiliations:** 1 Internal Medicine, Shalamar Hospital, Lahore, PAK; 2 Internal Medicine, Chughati Medical Centre, Lahore, PAK; 3 Internal Medicine, Sir Ganga Ram Hospital, Lahore, PAK; 4 Internal Medicine, Abwa Hospital, Khurrianwala, PAK; 5 Internal Medicine, Sheikh Zayed Hospital, Rahim Yar Khan, PAK

**Keywords:** gastrointestinal bleeding, intestinal perforation, salmonella paratyphi, salmonella typhi, typhoid fever

## Abstract

Background

Typhoid fever remains a major public health concern in endemic regions, characterized by diverse clinical manifestations and significant morbidity and mortality.

Objective

To evaluate the clinical spectrum, complications, and outcomes of patients with typhoid fever.

Methods

This retrospective study was conducted at Shalamar Hospital, Lahore, Pakistan, from April 2022 to April 2025. A total of 305 patients with confirmed typhoid fever were included in the study. Patients were diagnosed through blood cultures or serological testing. Demographic information, presenting features, laboratory parameters, complications, treatment modalities, and outcomes were documented using a structured proforma.

Results

The mean age of patients was 22.6 ± 11.4 years, with a male-to-female ratio of 1.4:1. Fever was universal in 305 (100%), followed by abdominal pain in 189 (62.0%), loss of appetite 132 (43.3%), headache 123 (40.3%), diarrhea 106(34.8%), and constipation 74 (24.3%). Hepatomegaly and splenomegaly were observed in 92 (30.1%) and 68 (22.3%) of cases, respectively. Blood cultures were positive in 238 (78.0%) of patients, with *Salmonella Typhi* identified in 196 (64.3%). Complications occurred in 84 (27.5%) of patients, most commonly gastrointestinal bleeding 32 (10.5%) and intestinal perforation 21 (6.9%). The majority of patients recovered 287 (94.1%), while morbidity persisted in 9 (3.0%), and mortality was recorded in 9 (2.9%), mainly due to perforation and septicemia.

Conclusion

Typhoid fever continues to affect young populations disproportionately and presents with a broad spectrum of clinical features. Although most patients recover with appropriate treatment, complications remain frequent and contribute to significant morbidity and mortality.

## Introduction

Enteric fever is an acute systemic illness caused by a gram-negative bacterium, *Salmonella enterica*, *Serovar typhi* or *paratyphi* [1]. The major characteristics of the disease are that it derails by the persistence of high fever and may incorporate other symptoms like, malaise, headache, anorexia, constipation, diarrhea and non-productive cough [2]. At present, enteric fever is an international health problem; 11-21 million cases of enteric fever are estimated globally annually, and these manifestations lead to 200,000 deaths. Typhoid fever is a systemic disease-causing infection caused by *Salmonella enterica*, *Serovar typhi*, as well as *Salmonella paratyphi *A, B, and C to a minor extent [3]. Although there has been a breakthrough in antimicrobial treatment and preventive measures, it is still one of the most urgent infections in the developing world. Acute kidney injury may be diagnosed in 11 20 million cases each year globally, causing over 150,000 deaths [4]. The heaviest burden occurs in South Asia, where the poor sanitation conditions, unsafe water supply, and low availability of health infrastructure are major factors in disease transmission [5]. Spreading epidemiological coverage is also evidenced in the reports of the disease in some African regions. The etiology of typhoid fever is the consumption of contaminated food or water, escape of the bacteria due to the gastric acidity, and invasion of the intestinal mucosa [6]. Once the epithelial barrier is overcome, the Salmonella infiltrates the circulation and gains access to the body as a whole, causing a long-lasting febrile infection [7]. It has an incubation period of 7 to 14 days, and the clinical signs and symptoms vary, according to the strength of bacterial overload, host immunity, and underlying diseases. Step-ladder fever, abdominal pain, hepatosplenomegaly, rose spots with relative bradycardia are classic [8]. Nonetheless, in hyper-endemic areas, there is an emergence of atypical or less severe manifestations, especially in children, which makes early recognition and proper management difficult [9]. There is a broad clinical spectrum of typhoid fever. Although in many cases patients manifest uncomplicated fever and gastrointestinal symptoms, there are examples when they develop chronic complications when treatment is not conducted soon. One of the most feared complications is intestinal perforation, which is accompanied by extremely high mortality in case surgical and medical measures are not timely [10].

The other complications involve hemorrhage, hepatitis, myocarditis, meningoencephalitis, and septicemia. Neurological manifestations, which are unusual, have also been identified as being responsible for poor outcomes [11]. Typhoid fever can masquerade as other prevalent febrile disorders in children, and thus it is regularly underdiagnosed and treated too late. Trends in antimicrobial resistance predominate over the results of typhoid fever [12]. Historically, chloramphenicol transformed treatment by revolutionizing the treatment industry in the mid-20th century, but antibiotic resistance broke out promptly, demanding a switch to ampicillin, cotrimoxazole, and more recently, fluoroquinolones [13]. Excessive and inappropriate use of these agents, especially in endemic countries, favored the emergence of multidrug-resistant (MDR) and extensively drug-resistant (XDR) strains [14]. Emerging XDR *S. typhi* resistances, which are resistant to virtually all oral antimicrobials, in South Asia have caused consternation among clinicians and public health professionals [15]. These resistant cases have been characterized by treatment failures, which were long in terms of fever clearance, and high complication rates. The last two outcomes depend on the sociodemographic and clinical factors as well. Prognosis is commonly aggravated by malnutrition, young age, delay of hospital presentation, and co-infections [16]. Limited resources with poor diagnostic capacity, unavailability of blood cultures in a timely manner, and overuse of clinical diagnosis also set back prompt initiation of proper therapy in resource-limited settings. Further, the economic cost of hospital stay and long-term sickness takes a huge toll not only on the poorer communities but also adds to the poor health and poor-rich cycle [17].

Objective

The main objective of the study is to characterize the clinical presentation, laboratory profile, antimicrobial treatment patterns, and short-term outcomes, including complications, length of stay, and mortality.

## Materials and methods

Study setting and duration

This retrospective study was conducted at Shalamar Hospital, Lahore, Pakistan, from April 2022 to April 2025. A total of 305 patients with confirmed typhoid fever were included in the study. Patients were selected through non-probability consecutive sampling, including all eligible cases presenting during the study period. We used the single-proportion formula with p=0.27, Z=1.96, and d=0.05: n = (1.96² × 0.27 × 0.73) / 0.05² ≈ 302.9 [18]. Rounding up and allowing minimal attrition, the target became 305, which matches the achieved sample size.

Inclusion and exclusion criteria

The study included patients of all age groups and both genders with a confirmed diagnosis of typhoid fever, established either through a positive blood culture for *S. typhi *or *S. paratyphi*, or a positive typhidot/other validated serological assay in the presence of compatible clinical features. Patients were excluded if they had incomplete medical records, concurrent infections that could confound the diagnosis (such as malaria, dengue, or tuberculosis), or if they left the hospital against medical advice before completing treatment.

Data collection

A structured proforma was used to record demographic characteristics (age, gender, residence), clinical features (fever, abdominal pain, diarrhea, constipation, headache, hepatosplenomegaly, rose spots), and laboratory parameters, including complete blood counts, liver function tests, and blood culture or serological results. Complications such as gastrointestinal bleeding, intestinal perforation, hepatitis, septicemia, and neurological manifestations were documented. Management strategies, including choice of antimicrobial therapy, duration of hospital stay, and requirement of surgical intervention, were recorded. Outcomes were categorized as recovery, morbidity with persistent complications, or mortality.

Statistical analysis

All data were entered and analyzed using the Statistical Package for the Social Sciences (SPSS) version 26. Continuous variables such as age and duration of illness were expressed as mean ± standard deviation. Categorical variables, including gender distribution, presenting complaints, complications, and outcomes, were summarized as frequencies and percentages. Associations between categorical variables were assessed using the Chi-square test, while continuous variables were compared using independent sample t-tests. A p-value of ≤0.05 was considered statistically significant.

## Results

A total of 305 patients with confirmed typhoid fever were included in the study. The mean age of patients was 22.6 ± 11.4 years, ranging from 3 to 60 years. Males were modestly predominant 178(58.4%), giving a ~1.4:1 male-to-female ratio. Baseline leukocyte profile was mostly normal 172 (56.4%), but leukopenia was frequent 88 (28.9%), a pattern typical of enteric fever. Elevated liver enzymes in almost a quarter 71 (23.3%) suggest hepatocellular involvement. Blood cultures were positive in 238 (78.0%) patients, with *S. typhi* in 196 (64.3%) and *S. paratyphi* in 42 (13.8%). The remaining 67 (22.0%) patients were diagnosed based on serological testing, with IgM positivity in 52 cases and IgG positivity in 15 cases, confirming recent or past infection (Table 1).

**Table 1:** Demographic Characteristics of Patients with Typhoid Fever (n = 305) This table summarizes the demographic and baseline laboratory characteristics of patients diagnosed with typhoid fever (n = 305). Age is presented both as mean ± standard deviation and in categorical groups. Hematological profiles include leukopenia, normal, and leukocytosis ranges. Liver enzyme elevation is defined by ALT/AST above institutional cut-offs. Blood culture positivity and organism distribution are presented as frequencies and percentages. Data are descriptive; no statistical comparison was performed in this table.

Variable	Category	n (%)
Age, years (Mean ± SD)		22.6 ± 11.4
Age group	≤20 years	142 (46.6%)
	21–40 years	112 (36.7%)
	>40 years	51 (16.7%)
Sex	Male	178 (58.4%)
	Female	127 (41.6%)
Baseline WBC count	Leukopenia (<4,000/mm³)	88 (28.9%)
	Normal (4,000–11,000/mm³)	172 (56.4%)
	Leukocytosis (>11,000/mm³)	45 (14.8%)
Liver enzymes	Elevated	71 (23.3%)
Blood culture (any positive)	—	238 (78.0%)
Organism among positives	S. typhi	196 (64.3%)
	S. paratyphi	42 (13.8%)
Serology	IgM positive	52 (17.0%)
	IgG positive	15 (5.0%)

Fever was universal 305 (100%), and gastrointestinal symptoms were prominent, with abdominal pain 189 (62.0%), followed by loss of appetite 132 (43.3%), headache 123 (40.3%), diarrhea 106 (34.8%), and constipation 74 (24.3%). Examination frequently revealed hepatomegaly in 92 (30.1%) and splenomegaly in 68 (22.3%), reflecting reticuloendothelial involvement (Table 2).

**Table 2:** Clinical Features of Typhoid Fever Patients (n = 305) This table outlines the clinical presentation of patients with confirmed typhoid fever. Fever was universal, while gastrointestinal symptoms such as abdominal pain, diarrhea, and constipation predominated. Organomegaly findings (hepatomegaly and splenomegaly) were based on clinical examination. All data are presented as n and percentages. This descriptive summary provides the clinical spectrum observed at admission.

Clinical Feature	Frequency (n)	Percentage (%)
Fever	305	100.0
Abdominal pain	189	62.0
Diarrhea	106	34.8
Constipation	74	24.3
Headache	123	40.3
Loss of appetite	132	43.3
Hepatomegaly	92	30.1
Splenomegaly	68	22.3
Blood culture: S. typhi	196	64.3
Blood culture: S. paratyphi	42	13.8

Over a quarter of patients developed complications 84 (27.5%), chiefly gastrointestinal bleeding 32 (10.5%) and intestinal perforation 21(6.9%), with additional cases of hepatitis 18 (5.9%), septicemia 9 (3.0%), and rare encephalopathy 4 (1.3%). Overall outcomes were favorable: 287 patients (94.1%) recovered fully, while morbidity 9 (3.0%) included patients who survived but had persistent complications requiring prolonged care. Mortality 9 (2.9%) occurred primarily in patients with severe presentations such as intestinal perforation or septicemia, often associated with delayed hospital presentation or comorbidities. Regarding therapy, ceftriaxone was the most commonly used agent 182 (59.7%), followed by azithromycin 63 (20.7%) and combination therapy 38 (12.5%), reflecting contemporary, resistance-aware practice. Fluoroquinolone use was limited to 14 (4.6%) due to reduced efficacy in endemic regions (Table 3).

**Table 3:** Complications, Outcomes, and Treatment Modalities in Typhoid Fever Patients (n = 305) This table presents the complication profile, outcomes, and treatment regimens used in typhoid fever patients. Complications include both gastrointestinal (bleeding, perforation) and systemic (hepatitis, septicemia, encephalopathy) events. Outcomes are categorized into recovery, morbidity, and mortality. Antimicrobial therapy distribution is shown, with ceftriaxone as the most frequently used agent. Data are descriptive; no inferential statistics are applied in this table.

Domain	Category	n (%)
Complications	Gastrointestinal bleed	32 (10.5%)
	Intestinal perforation	21 (6.9%)
	Hepatitis	18 (5.9%)
	Septicemia	9 (3.0%)
	Encephalopathy	4 (1.3%)
	Any complication	84 (27.5%)
Outcomes	Recovered	287 (94.1%)
	Morbidity	9 (3.0%)
	Mortality	9 (2.9%)
Treatment	Ceftriaxone	182 (59.7%)
	Azithromycin	63 (20.7%)
	Combination therapy (Ceftriaxone + Azithromycin)	38 (12.5%)
	Oral fluoroquinolone	14 (4.6%)
	Supportive care only	8 (2.6%)

Patients with complications were older on average (26.4 ± 10.8 vs 21.1 ± 11.2 years; p=0.04), suggesting age-related vulnerability or delayed presentation. Length of stay was substantially longer with complications (12.6 ± 4.3 vs 7.2 ± 2.1 days; p<0.001), indicating higher resource utilization. Mortality was confined to the complicated group 9(10.7%) vs 0(0%); p<0.001, underscoring the prognostic weight of complicated disease. Sex distribution did not differ meaningfully (p=0.63), implying that risk for adverse course is not primarily gender-driven (Table 4).

**Table 4:** Comparison of Patients With vs Without Complications (n = 305) This table compares demographic and outcome variables between patients with complications (n = 84) and those without complications (n = 221). Continuous variables (age, hospital stay) were analyzed using the independent samples t-test, while categorical variables (gender, mortality) were analyzed using the Chi-square test. Statistically significant differences (p ≤ 0.05) are marked with an asterisk (*). The analysis highlights that complicated cases were older, had longer hospital stays, and accounted for all observed mortality.

Variable	With Complications (n=84)	Without Complications (n=221)	p-value
Mean age (years)	26.4 ± 10.8	21.1 ± 11.2	0.04*
Male gender (%)	51 (60.7%)	127 (57.5%)	0.63
Mean hospital stay (days)	12.6 ± 4.3	7.2 ± 2.1	<0.001*
Mortality (%)	9 (10.7%)	0 (0.0%)	<0.001*

## Discussion

This study evaluated the clinical spectrum, complications, and outcomes of 305 patients with typhoid fever. The findings of this investigation prove that despite most of the patients recovering with adequate treatment, a fair percentage of them develop complications, and rather minor, yet considerable mortality burden remains. Demographic characteristics in this study revealed that the population of patients under 20 years was about half, the average age was found to be 22.6 years, and the men outnumbered the women. The distribution of this conforms with past studies that have indicated an over-concentration of typhoid fever among children, adolescents, and young adults. Its higher occurrence in men could be identified based on the high exposure to contaminated food and water sources because of workplace and outdoor activities [16]. These results are consistent with other existing studies, which unanimously state that the hallmark symptom of typhoid fever is long duration of fever and gastrointestinal symptoms [17]. Hepatomegaly and splenomegaly, as found in a large group of patients, are also reflective of the previous research since they indicate that reticuloendothelial involvement is a common phenomenon. This morphism in symptoms explains the high rate of misdiagnosis of typhoid fever in the endemic regions, where fevers such as malaria and dengue can resemble typhoid fever. Analysis exposed that most of the patients had leukopenia and raised liver enzymes [19]. This observation is similar to that in a prior study that has documented hematological abnormalities and biochemical disturbance in a large proportion of patients. The low itself of a high culture positivity rate in the study, and the dominance of *S. typhi* as the isolate, supports the further dominance of *S. typhi* as compared to the *S. paratyphi* in endemic sites. Nevertheless, the use of blood cultures only in a subsection of the population and including the serological tests also indicates the limitations typically met in a resource-poor setup [20]. The comparatively reduced incidence in this paper could be attributed to an enhancement in diagnosis ability and early therapeutic initiation. The occurrence of intestinal perforation and septicemia was significantly linked to mortality, and this is in line with other studies that have already highlighted this complication as an important predictor of poor outcome [21].

The general mortality rate of 2.9% in the study lies within the limits of other studies with a similar hospital managers, whereas the fatality level is recorded to be higher in community-based analyses because of a delay in getting treatment. Observation of treatment trends indicated that third-generation cephalosporins, in particular, ceftriaxone prevailed, and azithromycin and combined therapy were the most common agents. This is to reduce the ineffectiveness of fluoroquinolones due to the current trends in the endemic countries, due to resistance [22]. The use of cephalosporins and azithromycin reflects the changes in antibiotics described in earlier studies, but the development of new resistance risks is emerging as a serious problem. It was observed that the length of stay in hospitals and mortality in the case of patients with complications were much higher, which stresses the weight, essentially venerable, of resistant or progressive disease, justifying the necessity of early diagnosis and treatment [23]. Results in this procedure were on the whole positive, with 94.1% of patients recovering fully. Nonetheless, 3 percent experienced morbidity, and 2.9% died, with the highest characteristics limited to the development of severe complications. These results highlight that although most of the cases may be handled adequately, there is a group of patients who may be prone to severe consequences [24]. Gastrointestinal bleeding and manifestations of systemic toxicity are seen as warning signs that should be identified early to minimize morbidity and mortality. This research has consequences for public health as well. The relatively young mean age pattern helps to explain the necessity to launch specific vaccination campaigns, first of all with typhoid conjugate vaccines, as, according to the previous studies, they are very effective, particularly among children and teenagers. Also, the fact that the complications persisted even under the aftermath of antimicrobial treatment demonstrates that more powerful surveillance networks, resistance-surveillance, and the availability of culture labs in areas with the malady are required [25]. Control of underlying risk factors like unsafe water, poor sanitation, and poor food hygiene remains the prime long-term control of typhoid fever.

This study has several limitations. Its single-center, retrospective design limits the ability to establish causality and may reduce generalizability, particularly to community cases, which might present earlier and with milder symptoms. Selection bias may be present due to non-probability consecutive sampling, and tertiary-care referral patterns could overestimate complication rates. Diagnosis relied on both blood culture and serology (e.g., Typhidot), which may have led to misclassification, particularly in serology-positive but culture-negative patients. Prior antibiotic exposure could also reduce culture sensitivity. Furthermore, antimicrobial susceptibility data were not systematically collected for all patients, preventing meaningful analysis of the relationship between antibiotic resistance and morbidity or mortality.

## Conclusions

It is concluded that typhoid fever continues to pose a considerable health burden, particularly among children, adolescents, and young adults. The disease presents with a wide clinical spectrum, most commonly characterized by fever and gastrointestinal symptoms, and may progress to severe complications such as gastrointestinal bleeding, intestinal perforation, hepatitis, and septicemia. In this study, the majority of patients achieved recovery with appropriate antimicrobial therapy, yet a notable proportion developed complications, and mortality was primarily linked to advanced disease at presentation.

## References

[1] Herekar F, Sarfaraz S, Imran M, Ghouri N, Shahid S, Mahesar M (2022). Clinical spectrum and outcomes of patients with different resistance patterns of Salmonella enterica. Pak J Med Sci.

[2] Jog S, Soman R, Singhal T, Rodrigues C, Mehta A, Dastur FD (2008). Enteric fever in Mumbai--clinical profile, sensitivity patterns and response to antimicrobials. J Assoc Physicians India.

[3] Siddiqui FJ, Rabbani F, Hasan R, Nizami SQ, Bhutta ZA (2006). Typhoid fever in children: Some epidemiological considerations from Karachi, Pakistan. Int J Infect Dis.

[4] (2020). Centers for Disease Control and Prevention. Typhoid fever and paratyphoid fever [Internet]. Atlanta (GA): CDC. https://www.cdc.gov/typhoid-fever/health-professional.html.

[5] Gashaw T, Jambo A (2022). Typhoid in less developed countries: A major public health concern. IntechOpen.

[6] Chatterjee R, Chowdhury AR, Mukherjee D, Chakravortty D (2023). From Eberthella typhi to Salmonella typhi: The fascinating journey of the virulence and pathogenicity of Salmonella typhi. ACS Omega.

[7] Ogata T, Akakura K, Mizoguchi K, Mikami K, Nozumi K, Ito H (2003). Annual changes of the incidence and clinical characteristics of magnesium ammonium phosphate urinary stones. Int J Urol.

[8] Hoffner RJ, Slaven E, Perez J, Magana RN, Henderson SO (2000). Emergency department presentations of typhoid fever. J Emerg Med.

[9] Owais A, Sultana S, Zaman U, Rizvi A, Zaidi AK (2010). Incidence of typhoid bacteremia in infants and young children in southern coastal Pakistan. Pediatr Infect Dis J.

[10] Sharma A, Sharma R, Sharma S, Sharma A, Soni D (2013). Typhoid intestinal perforation: 24 perforations in one patient. Ann Med Health Sci Res.

[11] Marchello CS, Birkhold M, Crump JA (2020). Complications and mortality of typhoid fever: A global systematic review and meta-analysis. J Infect.

[12] Britto C, Pollard AJ, Voysey M, Blohmke CJ (2017). An appraisal of the clinical features of pediatric enteric fever: Systematic review and meta-analysis of the age-stratified disease occurrence. Clin Infect Dis.

[13] Azmatullah A, Qamar FN, Thaver D, Zaidi AK, Bhutta ZA (2015). Systematic review of the global epidemiology, clinical and laboratory profile of enteric fever. J Glob Health.

[14] Sarswat S, Kumar M, Gupta R (2018). Pediatric nature of enteric fever with emerging antibiogram: A cross sectional study. Int J Pediatr Res.

[15] Akram J, Khan AS, Khan HA, Gilani SA, Akram SJ, Ahmad FJ, Mehboob R (2020). Extensively drug-resistant (XDR) typhoid: Evolution, prevention, and its management. Biomed Res Int.

[16] Jorgensen Jorgensen, James H, and issuing body National Committee for Clinical Laboratory Standards (1995). Performance Standards for Antimicrobial Susceptibility Testing.

[17] Zakir M, Khan M, Umar MI, Murtaza G, Ashraf M, Shamim S (2021). Emerging trends of multidrug-resistant (MDR) and extensively drug-resistant (XDR) Salmonella typhi in a Tertiary Care Hospital of Lahore, Pakistan. Microorganisms.

[18] Muche G, Tesfaw A, Bayou FD (2024). Prevalence of typhoid fever and its associated factors among febrile patients visiting Arerti Primary Hospital, Amhara Region, north east Ethiopia. Front Public Health.

[19] Chatterji S, Sharma P, Kaur A (2022). A study on the clinical spectrum, prescription pattern and diagnosis of enteric fever in a tertiary care hospital of North India. Int J Commun Med Public Health.

[20] Shahid S, Mahesar M, Ghouri N, Noreen S (2021). A review of clinical profile, complications and antibiotic susceptibility pattern of extensively drug-resistant (XDR) Salmonella Typhi isolates in children in Karachi. BMC Infect Dis.

[21] Katiyar A, Sharma P, Dahiya S, Singh H, Kapil A, Kaur P (2020). Genomic profiling of antimicrobial resistance genes in clinical isolates of Salmonella Typhi from patients infected with Typhoid fever in India. Sci Rep.

[22] Sharma P, Dahiya S, Balaji V (2016). Typhoidal salmonellae: Use of multi-locus sequence typing to determine population structure. PLoS One.

[23] Casewell M (1979). Mackie and McCartney. Medical Microbiology. A Guide to the Laboratory Diagnosis and Control of Infection. J Clin Pathol.

[24] Muteeb G, Rehman MT, Shahwan M, Aatif M (2023). Origin of antibiotics and antibiotic resistance, and their impacts on drug development: A narrative review. Pharmaceuticals (Basel).

[25] Saeed DK, Hasan R, Naim M (2017). Readiness for antimicrobial resistance (AMR) surveillance in Pakistan; a model for laboratory strengthening.. Antimicrob Resistance Infect Control.

